# Editorial: Insights in cardiovascular biologics and regenerative medicine: 2022

**DOI:** 10.3389/fcvm.2023.1333866

**Published:** 2023-11-17

**Authors:** Paolo Madeddu, Ngan F. Huang

**Affiliations:** ^1^ Bristol Medical School, Translational Health Sciences and Bristol Heart Institute, University of Bristol, Bristol, United Kingdom; ^2^ Department of Cardiothoracic Surgery, Stanford University, Stanford, CA, United States; ^3^ Stanford Cardiovascular Institute, Stanford University, Stanford, CA, United States; ^4^ Department of Chemical Engineering, Stanford University, Stanford, CA, United States; ^5^ Center for Tissue Regeneration, Repair and Restoration, Veterans Affairs Palo Alto Health Care System, Palo Alto, CA, United States

**Keywords:** stem cells, fibrotic remodelling, vascular graft, aging, microRNA

**Editorial on the Research Topic** Insights in cardiovascular biologics and regenerative medicine: 2022

The cardiovascular Biologics and Regenerative Medicine field is rapidly evolving with recognition of new pathogenetic mechanisms and targets for treatment. One major challenge, however, is represented by the dichotomy between mechanistic studies and clinical applications. The goal of this special edition Research Topic is to shed light on the progress made in the past decade in the field of cardiovascular biologics and on its future challenges to provide a thorough overview of the field and to help moving on toward clinical studies.

The study from Adelino et al. elegantly assessed the safety profile of the adipose graft transposition procedure (AGTP). This approach consists of placing an autologous pericardial adipose pedicle over the infarct scar (1). It is believed that the adipose flap will continuously deliver mesenchymal stromal cells with immunomodulatory and angiogenic properties to the heart (2). This will be a more durable means to provide reparative cells to the chronically ischemic heart than injecting exogenous cells. The authors used ventricular endocardial high-density mapping and ECG-Holter monitoring for assessing the electrophysiological effects of the AGTP as compared with sham operation in a myocardial infarction (MI) swine model. Their findings indicate that AGTP is a safe reparative therapy in terms of arrhythmic risk and can exert protective effects against adverse electrophysiological remodeling in ischemic heart disease. These data are very important considering cell therapies have been accompanied by pro- and antiarrhythmic phenomena that could impact on their future clinical application. The study highlights the need of additional safety information that will hopefully emerge from the ongoing AGTP II randomized clinical trial (phase II-III; NCT02798276).

Another study with translational implications relates to tissue engineering of vascular grafts for potential bypass grafting for cardiac or vascular surgery. Engineered vascular grafts were initially described several decades ago (3), but the clinical adoption of this technology remains slow (4). Some of the technical hurdles include overcoming thrombosis by endothelialization of the inner lumen, providing structural integrity to resist failure, and withstanding physiological shear stress dynamics. To address some of these challenges, Rizzi et al. developed silk-based vascular grafts that take advantage of their strong mechanical properties, biocompatibility, and scalable manufacturing. To generate an endothelialized inner lumen, the authors utilized a dynamic cell seeding procedure based on low-speed rotation that supports uniform attachment of endothelial cells to the lumen and the provision of shear stress. While this rotational strategy has been utilized before (5), the authors build on previous work by developing quantitative measures to assess endothelial coverage of the lumen. Nevertheless, future work is necessary to condition the vascular grafts to withstand physiological arterial shear stress levels as well as *in vivo* validation. These findings highlight the complexity of native blood vessels and the design criteria to recapitulate their essential function in vascular grafts.

In the field of cellular biologics for the adjuvant treatment of myocardial ischemia, a number of cell types have been clinically tested for the treatment of myocardial infarction and heart failure, including stem cells, progenitor cells, and mononuclear cells derived from the bone marrow (6). Whereas prior work heavy focus on the effects of cell transplantation on left ventricular function, Gowdak et al. examined the efficacy of bone marrow-derived cell therapy to treat myocardial perfusion and endothelial dysfunction in patients undergoing coronary artery bypass graft (CABG) surgery. In this small clinical study known as MiHeart/IHD, patients with severe coronary artery disease who underwent CABG procedure were injected with autologous bone marrow-derived cells. This trial showed that cell therapy as an adjuvant to CABG was safe and improved regional stress-induced myocardial ischemia after 30 days, compared to placebo treatment. The authors postulated a potential mechanism of action being the paracrine release of extracellular vesicles. This work highlights the potential benefit of stem cell therapy for treating stress-induced myocardial ischemia in the setting of CABG and should be validated by longer term studies with mechanistic underpinnings.

Fibrotic remodelling of the ischemic and aging heart is a finely modulated phenomenon. Fibrosis replacement does not reconstitute the function of organs damaged by ischemic and metabolic injury. However, an efficient and solid scar can avoid severe complications, such as cardiac aneurism formation and rupture. After a plaque ulceration, the contraction of myofibroblasts together with re-endothelialization of the culprit artery can lead to plaque stabilization. A spectrum of novel mechanisms is currently investigated to drive fibrosis toward organ protection and damage stabilization. In addition to the classical renin-angiotensin system, (Pro)renin and its receptor (PRR) exert complementary control of fibrotic remodelling in many human diseases (7). In a review article of this thematic series, Wang et al. provide an extensive and accurate report of a research field that goes back to 2022 and is still open to effective pharmacological modulation. The authors highlight the pathophysiological role of the PRR and downstream pathway in the brain, where the PPR can have strong implications for arterial hypertension development. The soluble form of the PRR is seemingly implicated in obesity-associated hypertension and sodium retention at the kidney level. Despite treatments for arterial hypertension are well developed, a significant proportion of patients on multi-drug therapy do not reach recommended levels of blood pressure (8). Inhibiting the PRR could be helpful in reaching the target. The discovery and use of length bait peptide, called handle-region peptide (HRP), acting as a competitive inhibitor raised promises in some animal models, but clinical results were not convincing (9). The review article also focuses on the association of PRR, Erk1/2, Wnt/β-catenin signaling pathway, and the mechano-transducer YAP in the pathogenesis of myocardial fibrosis caused by metabolic disease. A novel antagonistic peptide, named PRO20, which is the first 20 amino acids of the (pro)renin pro-segment, seems to exert a potent inhibitory effect on the above pathogenic mechanism (10).

In recent decades, it is become well-appreciated that non-coding RNAs such as microRNAs (miRNAs) and long noncoding RNAs (lnc-RNAs) provide instructive cues that regulate physiological tissue function as well as disease pathology. Since non-coding RNAs regulate gene expression, they play key roles in cardiac aging and the progression of cardiovascular disease. This review article by Varghese et al. provides a summary of aging-associated molecular alterations to cardiovascular cell types that include telomeric shortening, epigenetic modifications, mitochondrial dysfunction, and cellular senescence (11). The signaling mechanisms by which the dysregulation of miRNAs and lnc-RNAs contribute to the development of aging-associated cardiovascular diseases is described, along with forward-thinking strategies to apply the fundamental knowledge for diagnostic or therapeutic purposes.

In conclusion, this thematic issue has the merit to underscore common pathogenetic mechanisms that can be targeted either pharmacologically or through novel tissue engineering solutions to foster endogenous repair. Organ reconstitution represents the optimal outcome, yet the less ambitious target of stabilizing the disease through modulation of fibrosis or cell therapy could result in tangible improvement of life quality and expectancy of patients with cardiovascular disease. Hopefully, this article collection will inspire, inform and provide direction and guidance to researchers in the field.
